# IGF1 and IGF2 specificities to the two insulin receptor isoforms are determined by insulin receptor amino acid 718

**DOI:** 10.1371/journal.pone.0178885

**Published:** 2017-06-01

**Authors:** Mie Andersen, Dorte Nørgaard-Pedersen, Jakob Brandt, Ingrid Pettersson, Rita Slaaby

**Affiliations:** Novo Nordisk, Novo Nordisk Park, Maaloev, Denmark; Russian Academy of Medical Sciences, RUSSIAN FEDERATION

## Abstract

**Methods:**

Alanine scan of insulin receptor (IR)-B exon 11 and site-directed mutagenesis of amino acid 718 in human IR-A and IR-B were performed. Ligand affinities to wild type and mutated receptors were studied by displacement of radioactive insulin in binding assay on secreted soluble midi receptors or solubilized semi-purified full length receptors stably expressed in Baby Hamster Kidney cells. Phosphorylation of IR in response to insulin, IGF1 and IGF2 was measured using ELISA.

**Results:**

Insulin, insulin detemir and insulin glargine maximally showed two fold differences in affinity for human IR-A and IR-B, but IGF1 and IGF2 had up to 10 fold preference for IR-A. Alanine scan of exon 11 revealed that position 718 is important for low IGF1 affinity to IR-B. Mutational analysis of amino acid residue 718 in IR-A and IR-B demonstrated that charge is important for IGF1 and IGF2 affinity but not important for insulin affinity. The affinity of IGF1 and IGF2 for the mutant IR-A P718K was comparable to the wild type IR-B whereas the affinity of IGF1 and IGF2 for the mutant IR-B K718P was comparable to the wild type IR-A. Changes in affinity were also reflected in the IR activation pattern.

**Conclusion:**

Mutating position 718 in human IR-B to the proline found at position 718 in human IR-A increased IGF1 and IGF2 affinity to a level comparable to IR-A and mutating position 718 in IR-A to the lysine found at position 718 in IR-B decreased IGF1 and IGF2 affinity to a level comparable to IR-B, whereas a negatively charged glutamate did not. These changes in the affinities were also reflected in the IR phosphorylation pattern, meaning that position 718 is important for both affinity and activation of the receptor. It should be emphasized that none of the mutations affected insulin affinity, indicating that the mutations did not alter the overall receptor structure and that the effect is ligand specific.

## Introduction

The human insulin receptor (IR) is a ligand activated transmembrane receptor tyrosine kinase that exists as two isoforms [1] [2]. It is a glycoprotein transcribed from one gene encoding theα and β subunits. Cleavage and dimerization lead to the mature IR which has a covalently dimeric structure consisting of two α subunits and two α subunits [3]. The two IR isoforms differ by the absence (IR-A) or the presence (IR-B) of the exon 11 encoded 12 amino acids at the C-terminal part of the α subunit and the relative isoform abundance is regulated at the pre-mRNA level by alternative splicing of exon 11 [4]. The ratio between the two isoforms of IR varies between different tissues [5] [6], where IR-A is highly expressed in human kidney and brain, while IR-B is the predominant isoform in human liver [7]. Alternative splicing is hormonally regulated and is altered during development and under a number of pathological conditions such as type 2 diabetes and cancer [8]. IR regulates glucose metabolism and cell growth, but the functional role of having two IR isoforms is not fully understood. Insulin-like growth factor 2 (IGF2) has high affinity for the IR-A isoform, being only five fold lower than insulin affinity to IR-A. Since the total level of IGF2 in human blood is approximately 1 mg/l [9], IGF2 should be considered as an important ligand for IR-A.

The difference in insulin affinity for IR-A and IR-B is at most two fold [10], but the two hormones insulin-like growth factor 1 (IGF1) and IGF2 have demonstrated up to five fold higher affinity to IR-A than IR-B, although IGF1 binding is relatively weak compared to insulin and IGF2 binding [11] [12] [13] [14]. IGF1 has higher affinity to the human insulin-like growth factor 1 receptor (IGF1R) and to hybrid receptors formed between IR and IGF1R than homodimer IR [12] [15]. The IGFs promote cell growth, survival, migration and differentiation [16] and the level of free IGFs in the blood is highly regulated by the IGF2/mannose-6-phosphate receptor and IGF binding proteins (IGFBPs) [17].

**Fig 1:**
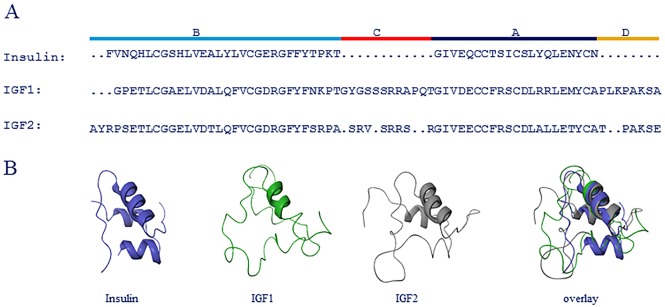
Comparison of insulin, IGF1 and IGF2 structure. (A) Sequence alignment of insulin, IGF1 and IGF2. Different protein regions are indicated at the top. (B) Comparison of folded structure of insulin (blue), IGF1 (green) and IGF2 (gray).

Insulin belongs to the superfamily of insulin related proteins that include IGF1 and IGF2 [18] [19] [20]. Pro-insulin consists of three domains; A, B and C, where the C domain is cleaved off upon maturation. There are several splice variants of IGF1 which have different affinities to IGF1R [21]. This study examines mature insulin which is a two-chain peptide consisting of the A and B domains and mature IGF1 and IGF2 which are single-chain molecules consisting of the four domains A, B, C and D (Fig 1). The C-domain is not cleaved off from IGF1 and IGF2 upon maturation as it is from the insulin molecule.

Mutational analysis of IGF2 suggests that IGF2 and insulin have similar binding surfaces but subtle differences could contribute to IR and IGF1R specificities; however, these binding surfaces do not contribute to the selectivity between IR-A and IR-B [22]. The C-domain of IGF2 is important for increased activation of IR-A compared to IR-B [23] and the mechanism by which IGF1 and IGF2 activate IR and IGF1R is similar to insulin activation of IR by cross bridging the two receptor halves [24].

Crystal structure analysis of truncated IR/IGF1R hybrids in complex with IGF1 [25] suggest that the C-terminal part of the IR α subunit, denoted αCT peptide, is located inside the loop of IGF1. The 12 amino acid extension encoded by exon 11 in IR-B would have to thread through or somehow point away from the IGFs’ C-loop, hence the lower IGF1 and IGF2 affinity to IR-B could be explained by steric hindrance between the IGFs and exon 11 encoded amino acids [25]. The selectivity of IGF2 for IR-A may be important in cancer and cancer treatment since some cancer cells express increased level of IR-A in combination with increased IGF2 expression [26].

The functional binding epitopes of IR-A and IR-B have been studied by mutational analysis such as alanine scanning [27] but until now the exon 11 encoded sequence has been excluded. We have examined the difference between IR-A and IR-B in the capability to bind insulin, insulin detemir, insulin glargine, IGF1 and IGF2. Compared to insulin binding, we found a balanced binding of insulin detemir and insulin glargine, whereas IGF1 and IGF2 had up to 10 fold preference for IR-A. Mutational analysis revealed that amino acid 718 in IR-A and IR-B is important for IGF1 and IGF2 affinity, but not for insulin affinity. IGF affinities could be either increased or decreased by mutating position 718 in IR without affecting insulin affinity. This position is therefore important for the differential affinity of IGF1 and IGF2 to the two IR isoforms.

It has been reported that IGF2 binds to IR-A with high affinity and that IGF2 stimulation of IR-A leads to more mitogenic signaling than insulin stimulation [14] [28], however, it remains unanswered whether this is solely due to the high IGF2 affinity to IR-A or whether the IR-A/ IGF2 complex intrinsically signals more mitogenically.

IR activation can be measured as total tyrosine phosphorylation or by site-specific phosphorylations in three different parts of IR β-subunit; juxtamembrane domain, kinase domain and carboxy-terminus. The EC50 value, the maximal response and the kinetics of IR activation are reported to be ligand dependent [29]. Other sources report ligand-dependent differential phosphorylation of the three different IR phosphorylation areas [30], which might influence signaling downstream from IR. We found a correlation between the affinity for the receptors and receptor activation measured as total level of tyrosine phosphorylation of the receptor.

## Materials and methods

### Cells and culture conditions

Wild type (WT) human IR-A, IR-B and mutated IRs were inserted in pZem vector and stably over-expressed in Baby Hamster Kidney (BHK) cells to achieve the desired receptor expressions. In brief, 1 x 10^6^ cells were transfected with 10 μg plasmid by liposome transfer using Lipofectamine (Life Technologies, Tåstrup, Denmark) and allowed to recover three days before applying selection pressure in the form of 1 μM methotrexate. After approximately three weeks single cell clones from each transfection appeared and 12 clones from each transfection were picked and subjected to IR expression analysis by Western Blotting. Based on the highest expression level one clone for each transfection was selected and used in the subsequent experiments. Cells were cultured in Dulbecco’s modified Eagle medium containing high glucose, 10% fetal calf serum and 2 mM L-glutamine, 10 mg/ml streptomycin, and 100 units/ml penicillin (Invitrogen, Nærum, Denmark) at 37°C in a 5% CO_2_ enriched, humidified atmosphere.

Midi receptor mutants of human IR were made in the soluble high affinity midi receptor construct [31], but with an intact functional furine processing site followed by a small part of the β-subunit giving rise to a genuine IR C-terminal alpha subunit sequence without tags. Two unique restriction sites (*AvrII* and *BstB*I) were made to frame exon 11 without changing the amino acid sequence. Oligos with the desired mutations were designed to include the *AvrII* site (upstream of exon 11). An oligo downstream of exon 11 and the *BstB1* was used to obtain a PCR product which was subcloned, hereby replacing the *AvrII*/*BstBI* fragment in human IR-A or human IR-B. The point mutation at position 718 in full-length receptors included IR-A P718A, P718E and P718K and IR-B K718A, K718E and K718P.

### Chemicals and antibodies

^125^I-(Tyr31)-Insulin (^125^I-Insulin), human insulin, insulin determir, insulin glargine and human IGF1 were from Novo Nordisk A/S (Bagsvaerd, Denmark). Human IGF2 was from GroPep, Adelaide, Australia. The IR specific antibody 83–7 [32] was licensed from Professor K. Siddle, University of Cambridge, UK. HRP-conjugated anti-phosphotyrosine antibody 4G10 was obtained from Merck Millipore (cat. no. 16–316).

### WGA purification of solubilized IR

BHK cells over-expressing either WT or mutated human IR were lysed in 50 mM Hepes pH 8.0, 150 mM NaCl, 1% Triton X-100, 2 mM EDTA and 10% glycerol. The cleared cell lysate was batch absorbed with wheat germ agglutinin (WGA)-agarose (Lectin from Triticum vulgaris-Agarose, L1394, Sigma-Aldrich Steinheim, Germany) for 90 minutes. The receptors were washed with 20 volumes 50 mM Hepes pH 8.0, 150 mM NaCl and 0.1% Triton X-100, where after the receptors were eluted with 50 mM Hepes pH 8.0, 150 mM NaCl, 0.1% Triton X-100, 0.5 M n-Acetyl Glucosamine and 10% glycerol. All buffers contained Complete (Roche Diagnostic GmbH, Mannheim, Germany).

### Scintillation proximity assay (SPA) binding assay

SPA PVT anti-mouse beads (Perkin Elmer) were diluted in SPA binding buffer, consisting of 100 mM HEPES, pH 8.0, 100 mM NaCl, 10 mM MgCl_2_, 0.025% Tween-20 and 0.05% BSA (Sigma A-6003). SPA beads were incubated with the IR-specific antibody 83–7 and either supernatant from BHK cells expressing midi receptors or solubilized semi-purified full length receptors for one hour at room temperature under gentle rocking. Receptor concentrations were adjusted to achieve 10% binding of ^125^I-Insulin.

SPA beads/receptor mixture was washed three times to remove IGFBPs. Dilution series of cold ligands in triplicates (insulin, IGF1 or IGF2) were added to 96 well Optiplate, followed by tracer (^125^I-Insulin, 5000 cpm/well) and lastly receptor/SPA mix. The plates were rocked gently for 22.5 hours at room temperature, centrifuged for 5 minutes at 850 x g and counted in TopCounter (Perkin Elmer).

### IR receptor activation

BHK cells stably over-expressing human IR-A, IR-B, IR-A P718K or IR-B K718P were starved for 1 hour, stimulated with insulin, IGF1 or IGF2 and incubated at 37°C and 5% CO_2_ for appropriate time. Cells were transferred to ice bath and washed with ice-cold phosphate buffered saline (PBS) (Gibco) containing protease and phosphatase-inhibitors to terminate the stimulation. The cells were lysed with lysis buffer (Cell Signaling) containing protease and phosphatase-inhibitors. The lysates were cleared by spinning 10 minutes at 14000 rpm and 4°C.

### Sandwich ELISA to measure total IR phosphorylation

Microtiter 96 well plates were coated with 100 μl 2 ug/ml 83–7 antibody per well for 1 hour at room temperature. Plates were washed three times with 200 μl PBS + 0.05% tween, blocked by adding three times 200 μl StartingBlock (Pierce). Cell lysates were added and incubated for 2 hours at 22°C and 400 rpm shake (iEMS incubator/shaker, Thermo Fisher). After 3 washes with PBS + 0.05% Tween 100 μl 4G10 antibody diluted 1:1000 in PBS was added to each well and incubated for two hours at 22°C and 400 rpm shake. After five washes with PBS + 0.05% Tween100 μl TMB substrate (Kem-En-Tec Diagnostics) was added to each well and incubated for 10 minutes, the reaction was stopped by addition of 50 μl 2M H_2_SO_4_ (Fisher Scientific) to each well and absorbance at 450 nm were determined using an ELISA reader (SpectraMax190, Molecular Devices).

### Alignment, calculations and statistics

All IC50 values and EC50 values were calculated using non-linear regression algorithm in GraphPad Prism 5.0 (GraphPad Software Inc., San Siego, CA). The geometric means with their corresponding 95% confidence intervals of at least three independent experiments were calculated. Two-tailed Student’s t-test was used for single statistical comparisons and one-way analysis of variance (ANOVA) with Dunnet’s correction for multiple comparisons was used for statistical testing in experiments where multiple treatments were analyzed. For all statistical analysis p<0.05 was considered statistically significant. For the IR displacement and activation curves representative examples are shown as mean with standard error of the mean (SEM) for triplicate measurements.

The alignment was done using the Protein Structure Alignment tool in Maestro version 10.6.014 [33]. The crystal structure of human insulin in T conformation (1MSO) [34], and NMR structures of IGF1 [35] IGF2 (1IGL) [20] were used in the alignment.

## Results

Affinity of insulin, insulin detemir, insulin glargine, IGF1 and IGF2 to solubilized full length human IR-A and IR-B receptors was measured using SPA binding assay with displacement of ^125^I-insulin. The geometric mean and 95% confidence interval were determined for each ligand to IR-A and IR-B, Table 1. Insulin affinity was in the low picomolar (pM) range and no significant differences were observed between IR-A and IR-B when comparing insulin to the two long acting insulin analogues insulin detemir and insulin glargine, analyzed by T-test (p<0.05). This was in contrast to IGF1 and IGF2, which were found to have at least 5 fold higher affinities to IR-A compared to IR-B, which is in agreement with the literature [36].

**Table 1:** Ligand affinities to IR-A and IR-B.

Cold ligand	IR-A IC50 (pM) Geometric mean (95% confidence intervals)	IR-B IC50 (pM) Geometric mean (95% confidence intervals)
**Insulin**	18.4 (14.9 to 22.7)	22.7 (19.6 to 26.1)
**Insulin detemir**	114 (52 to 248)	84.6 (29.1 to 246.0)
**Insulin glargine**	29.7 (15.9 to 55.4)	33.9 (26.1 to 43.9)
**IGF1**	863 (621 to 1199)*	4570 (2447 to 8536)
**IGF2**	95.3 (48.3 to 187.8)*	481 (251 to 920)

Affinity of human insulin, insulin detemir, insulin glargine, IGF1and IGF2 to full length human IR-A, IR-B was determined in SPA binding assay with displacement of ^125^I-insulin. The geometric mean and 95% confidence interval are given for independent experiments (n = 3 for all ligands except insulin n = 6).

*) Significant difference between IR-A and IR-B binding analyzed by T-test (p<0.05).

Aiming to identify the amino acids contributing to the low IGF1 and IGF2 affinity to IR-B, we performed an alanine scan of the amino acids encoded by exon 11. We performed the alanine scan in the midi receptor construct, which is described in [31]. The midi receptors are soluble by nature and secreted from the cells, hence binding experiments can be performed directly on media from the cells. After determination of the volume needed for 10% binding, the binding experiments were performed with ^125^I-insulin displacement of either insulin or IGF1.

**Fig 2:**
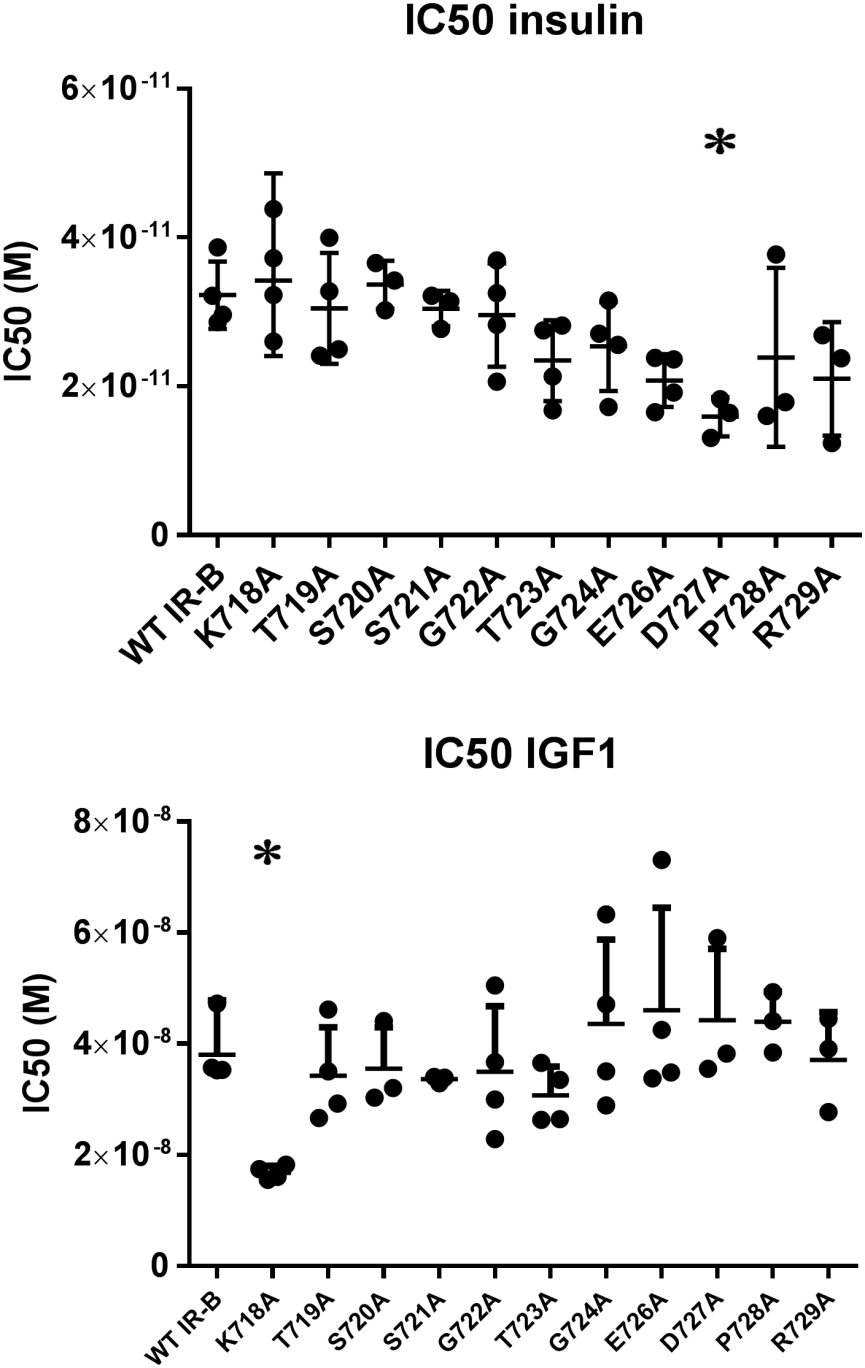
Position 718 is important for low IGF1 affinity to IR-B without influencing insulin affinity. IC50 values for insulin and IGF1 to WT IR-A and WT and exon 11 point-mutated IR-B midi receptors obtained in SPA binding assay with displacement of ^125^I-insulin for n = 3–4 independent experiments. *) Significant difference (p < 0.05) determined by ANOVA with Dunnet’s correction test.

No significant difference in insulin affinity was found between IR-A and IR-B, whereas the IGF1 affinity was significantly higher to IR-A compared to IR-B (Fig 2). Mutating position 727 (IR-B D727A) lead to a significantly increased insulin affinity without affecting IGF1 affinity, whereas none of the other mutations lead to any significant changes in insulin affinity. Mutating position 718 (IR-B K718A) lead to significantly increased IGF1 affinity compared to WT IR-B (Table 2).

**Table 2:** IC50 (pM) of insulin and IGF1 to exon 11 point-mutated IR-B midi receptors.

Receptor	Insulin IC50 (pM) Geometric mean (95% confidence intervals)	IGF1 IC50 (pM) Geometric mean (95% confidence intervals)
WT IR-A	25.2 (19.8 to 32.0)	3621 (3024 to 4334)
WT IR-B	32.0 (25.8 to 40.0)	38053 (30252 to 47865)
IR-B K718A	34.2 (24.0 to 48.6)	16735 (14882 to 18821)*
IR-BT719A	29.8 (20.4 to 43.6)	33478 (22708 to 49325)
IR-B S720A	33.5 (26.4 to 42.7)	34962 (21133 to 57842)
IR-B S721A	30.4 (24.9 to 37.0)	33600 (32195 to 35067)
IR-B G722A	28.9 (19.4 to 43.1)	33534 (19647 to 57236)
IR-B T723A	22.9 (15.6 to 33.9)	30352 (23198 to 39712)
IR-B G724A	24.8 (16.4 to 37.3)	41652 (24094 to 72004)
IR-B E726A	20.5 (15.5 to 27.2)	43732 (24770 to 77210)
IR-B D727A	15.8 (10.3 to 24.1)*	43098 (21814 to 85147)
IR-B P728A	22.1 (7.0 to 70.4)	43685 (32009 to 59623)
IR-B R729A	19.9 (7.1 to 56.1)	36352 (19775 to 66826)

The affinities of insulin and IGF1 to IR-A, IR-B and IR-B exon 11 point-mutated midi receptors were determined in SPA binding assay with displacement of ^125^I-insulin. The geometric mean of IC50 (pM) and the 95% confidence intervals are given for n = 3–4 independent experiments.

*) Significant difference (p < 0.05) determined by ANOVA with Dunnet’s correction test.

Since we aimed at finding positions important for IGF1 and IGF2 affinity without affecting insulin affinity, we focused on position 718. Four receptors for each isoform were made; WT IR-A, IR-A P718A, IR-A P718E and IR-A P718K and WT IR-B, IR-B K718A, IR-B K718E and IR-B K718P. These mutations were chosen in order to test the effect of introducing neutral, positive or negative amino acids and to test the impact of introducing a structural important amino acid. Stable BHK cell clones over-expressing the relevant receptor subtype were established and the receptors subsequently isolated, solubilized and semi-purified using WGA. Receptor expression levels were similar between clones indicating that none of the mutations changed receptor processing or folding (data not shown).

**Fig 3:**
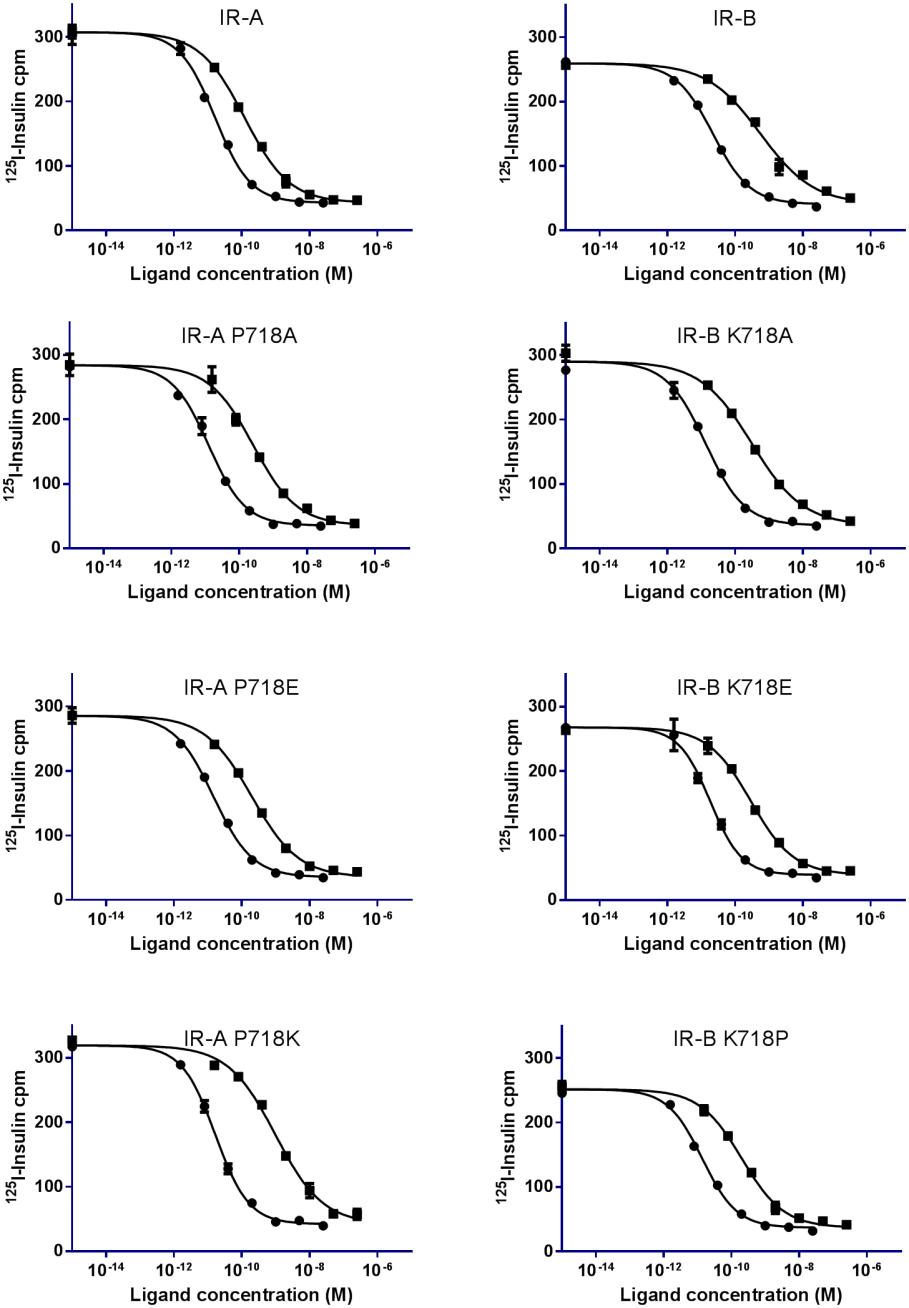
Displacement curves of ^125^I-insulin from WT IR and IR 718 mutants. A representative binding curve for WT IR-A, IR-A P718A, IR-A P718E and IR-A P718K and WT IR-B, IR-B K718A, IR-B K718E and IR-B K718P displacement of ^125^I-insulin with insulin (●) or IGF2 (■) is shown. Each point is the mean with SEM of triplicates. The experiments were performed three to five independent times.

Affinity was measured in SPA binding assay (Fig 3) and the affinities of mutated receptors were compared to corresponding WT receptors and analyzed by T-test (p<0.05). No significant difference in insulin affinity was observed between any of the receptors (Table 3)

**Table 3:** IC50 (pM) of insulin, IGF1 and IGF2 to position 718 mutated full length receptors.

Receptor	Insulin IC50 (pM) Geometric mean (95% confidence intervals)	IGF1 IC50 (pM) Geometric mean (95% confidence intervals)	IGF2 IC50 (pM) Geometric mean (95% confidence intervals)
**IR-A**	16.0 (7.2 to 35.6)	705 (363 to 1371)	95 (48 to 188)
**IR-A P718A**	15.1 (9.8 to 23.4)	1374 (1065 to 1773)	218 (151 to 316)
**IR-A P718E**	20.2 (8.9 to 45.9)	1059 (799 to 1404)*	154 (83 to 282)*
**IR-A P718K**	16.3 (10.2 to 26.0)	3672 (1591 to 8474)*	595 (240 to 1475)*
**IR-B**	13.0 (9.8 to 17.3)	4360 (2540 to 7486)	477 (259 to 877)
**IR-B K718A**	15.2 (7.4 to 31.1)	3622 (1603 to 8185)	288 (160 to 517)*
**IR-B K718E**	12.8 (10.1 to 16.7)	1496 (831 to 2693)*	225 (123 to 411)*
**IR-B K718P**	11.9 (7.6 to 18.7)	927 (429 to 2005)*	155 (78 to 311)*

The affinities of human insulin, IGF1 and IGF2 to IR-A, IR-B and position 718 mutated full length receptors were determined in SPA binding assay with displacement of ^125^I-insulin. The geometric mean of IC50 (pM) and the 95% confidence intervals are given for n = 3–5 independent experiments.

*) Significant difference (p < 0.05) determined by T-test between WT and mutant.

The mutational analysis showed that by changing the amino acid at position 718 between IR-A and IR-B, the IGF1 and IGF2 affinities were changed as well. The IC50 values for WT IR-A was 705 pM and 95 pM for IGF1 and IGF2, respectively, whereas for WT IR-B it was 4360 pM and 477 pM, respectively. By inserting the lysine found at position 718 in IR-B at position 718 in IR-A (IR-A P718K) the IC50 values were increased to 3672 pM for IGF1 and 595 pM for IGF2, which is significant different from WT IR-A but not significantly different from WT IR-B. IR-A P718E also significant decreased the affinity but IR-A P718A did not. By inserting the proline found at position 718 in IR-A at position 718 in IR-B (IR-B K718P) the IC50 values were decreased to 927 pM for IGF1 and 155 pM for IGF2, which is significantly different from WT IR-B, but not significantly different from WT IR-A. A significantly decreased IGF1 IC50 value were observed for IR-B K718E compared to WT IR-B, whereas IR-B K718A was not significantly decreased. All mutated IR-B receptors has significantly decreased IGF2 EC50 values compared to WT IR-B, meaning that all mutations significantly increased IGF2 affinity compared to IR-B (Table 3).

**Fig 4:**
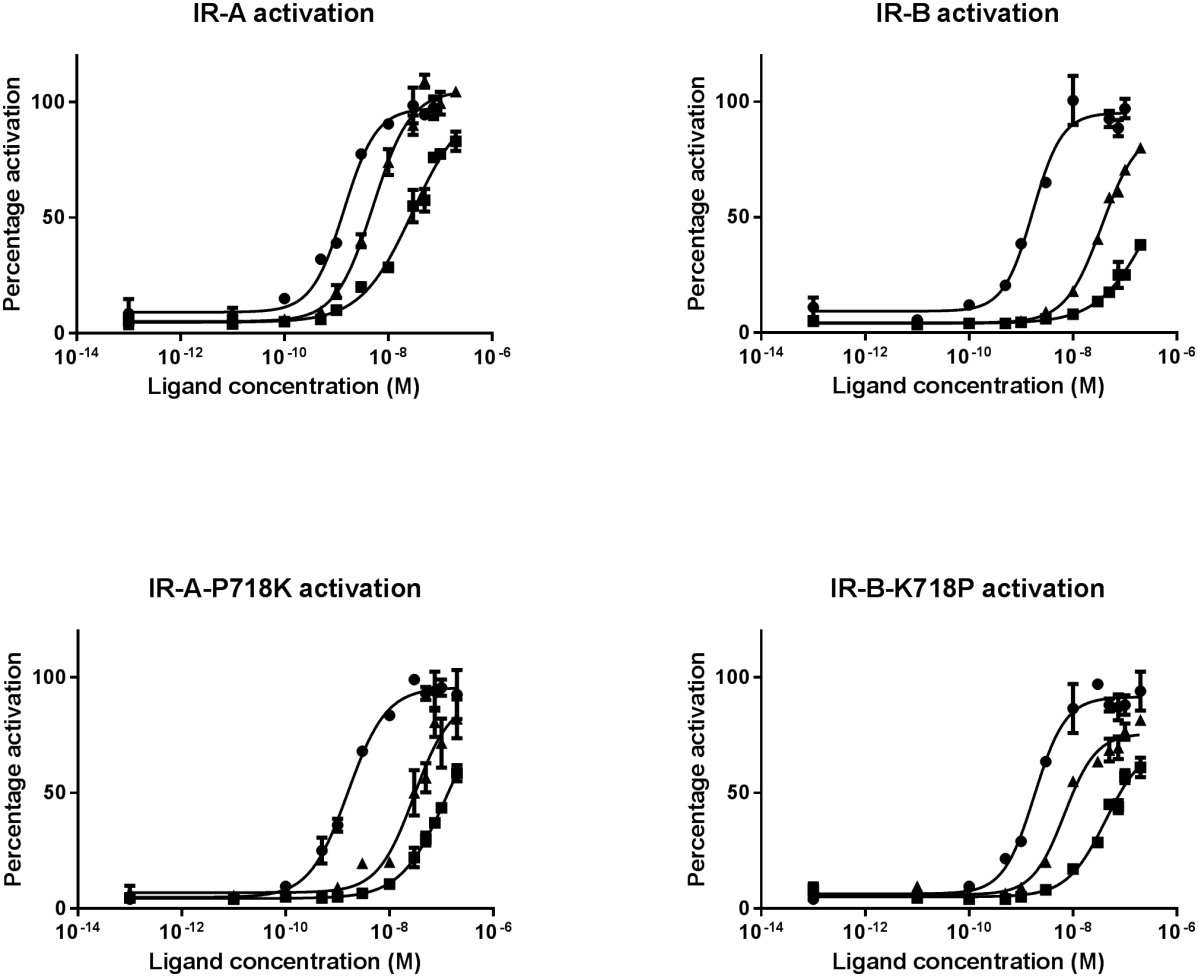
IR activation curves. A representative IR activation curve of IR tyrosine phosphorylation measured in sandwich ELISA after stimulation with insulin (●), IGF1 (■) or IGF2 (▲) in full concentration-response curves. Each data point is three measurements with SEM. The experiments were performed three independent times.

Finally we aimed at measuring whether changes in affinity translate into changes in IR activation, hence whether IR activation is mainly reflected by affinities or whether any of the ligands and/or receptors harbors some intrinsic differences in activation potential. This analysis included four receptors; WT IR-A, WT IR-B, IR-A P718K which is an IR-A receptor with IR-B like IGF1 and IGF2 affinities and IR-B K718P which is an IR-B receptor with IR-A like IGF1 and IGF2 affinities. IR activation was measured as total tyrosine phosphorylation in ELISA and time course studies revealed that IR activation was maximal or close to maximal for all receptors and ligands at 10 minutes stimulation time (data not shown), hence 10 minutes stimulation time was used for full concentration-responses curves. Representative full concentration-response curves are shown in Fig 4.

No significant differences in insulin EC50 values were observed between the mutated and the corresponding WT receptor, hence the mutations did not affect IR activation in response to insulin. IGF1 and IGF2 showed a significantly higher EC50 value for IR-A P718K compared to IR-A, meaning that IGF1 and IGF2 activate IR-A P718K significantly less efficiently than they activate IR-A. IGF1 and IGF2 showed a significantly lower EC50 value for IR-B K718P compared to IR-B, showing that IGF1 and IGF2 activate IR-B K718P significantly better than they activate IR-B. Table 4 summarizes the EC50 values calculated as geometric mean and 95% confidence intervals.

**Table 4:** EC50 values (nM) for IR activation and relative activation compared to IR-B.

Receptor	Insulin EC50 (nM) Geometric mean (95% confidence intervals)	IGF1 EC50 (nM) Geometric mean (95% confidence intervals)	IGF2 EC50 (nM) Geometric mean (95% confidence intervals)
**IR-A**	1.3 (0.4 to 4.1)	37 (11 to 123)	5.0 (4.5 to 5.6)
**IR-A P718K**	1.3 (0.4 to 4.2)	97 (53 to 177)*	23 (10 to 50)*
**IR-B**	1.8 (0.7 to 4.8)	308 (154 to 613)	32 (10 to 104)
**IR-B K718P**	1.7 (0.8 to 3.4)	68 (14 to 340)*	8.0 (5.2 to 12.1)*

IR activation measured as total tyrosine phosphorylation in ELISA assay for IR-A, IR-B, IR-A P718K and IR-B K718P. The geometric mean of EC50 (nM) and the 95% confidence intervals are given for n = 3 independent experiments.

*) Significant difference (p < 0.05) determined by T-test between WT and mutant.

The decrease in IGF2 affinity for IR-A with P718K is reflected in decrease in activation of the receptor, likewise the increase in IR-B affinity by the K718P mutation is reflected in the activation of the mutated receptor. The relation between affinity and IR activation does not apply as strictly for IGF1; however, this might be due to the very low affinity of IGF1 or assay-related challenges in measuring very low affinity and activation correctly.

## Discussion

The biological function of having two IR isoforms is not fully understood. Insulin and insulin analogues maximally show two fold differences in the affinity for IR-A and IR-B. However, a significant difference between the two IR isoforms is that IGF1 and IGF2 have fivefold higher affinities for IR-A compared to IR-B. The selectivity of IGF2 for IR-A may be important in cancer and cancer treatment since many cancer types overexpress IR-A and secretes IGF2 [26]. The amino acids encoded by exon 11 (position 718–729) are the only differences between IR-A and IR-B and these 12 amino acids have never been subjected to mutational analysis before. Data obtained in this study show that the amino acid at position 718 in IR is a major determinant of the IGF1 and IGF2 selectivity between IR-A and IR-B. Mutating position 718 can change the affinities of IGF1 and IGF2 to the receptor independent of the rest of the amino acids encoded by exon 11 without affecting insulin affinity.

Position 718 is the first amino acid in exon 11 and maps to the IR αCT peptide, which is defined as residues 704–719 in IR-A. The αCT peptide has been shown to be a part of the ligand binding site 1 through mutational analysis, and is proposed to interact directly with insulin, IGF1 or IGF2 [37], hence it seems plausible that this position contributes to IGF1 and IGF2 binding characteristics. Previous analyzes have mostly been focused on IR-A, where the α subunit ends with residue 719 whereas less is known about how the exon 11 extension of the α subunit in IR-B influences IR ligand binding site 1.

The crystal structure of IR-B has not been determined, hence the three dimensional position of the 12 amino acids encoded by exon 11 is unknown both in the apo-receptor and in the ligand bound state. Truncated versions of IR-A and IR-A/IGF1R hybrid receptors have been crystalized and from these structures it has been proposed how insulin, IGF1 and IGF2 interacts with the receptors. The αCT is displaced from IR upon ligand binding and insulin, in which the B-chain opens as a hinge upon engagement to IR [38]. The increased size of IGF1 and IGF2 compared to insulin together with the positioning of the C-region could lead to some steric hindrance when attempting to engage IR [25], since the C-region might reduce the flexibility of the IGF1 and IGF2 molecules. The flexibility of insulins B-region to open as a hinge has been claimed to be important for engagement of insulin to the IR [38], but opening of the IGF B-region would not be possible due to the C-region that connects the A- and B-region. It therefore seems likely that the reduced flexibility of IGF1 and IGF2 influences their ability to bind to IR.

It has been speculated that the position of αCT gives lower affinity to IR-B, because the 12 amino acids encoded by exon 11 extend αCT compared to in IR-A and that this extended αCT cannot pass through the loops in IGF1 and IGF2. Here we find that introducing K718P in IR-B results in high affinity and removing a P718 in IR-A results in low affinity. That can be explained by a structural change in the receptor backbone, where proline introduces a kink in the structure, hereby relocating the 12 amino acids so they thread away from the binding site. However, it is not only the backbone which is important. The charge of the amino acid at position 718 is important as well, since introducing a positively charged amino acid at position 718 in IR-A decreased IGF1 and IGF2 affinity whereas introducing a negatively charged amino acid at position 718 in IR-B increased IGF1 and IGF2 affinity. These observations suggest that differential IGF affinity between IR-A and IR-B is not only due to the structural features introduced by proline, but also due to electrostatic repulsion between positive charges in the IGFs C-domain and at position 718 in IR. Since only the amino acid at position 718 was found to influence IGF affinity to IR, these observations question whether the entire αCT protrudes through the loops of IGF1 and IGF2.

We found that IR activation mainly reflects affinities and that the specific IR isoform or specific ligand is of less importance. IR-A P718K is an IR-A receptor with decreased IGF1 and IGF2 affinity and this receptor also has decreased activation level in response to IGF1 and IGF2 in the same range as IR-B. Mutating IR-B with K718P is an IR-B receptor with increased IGF1 and IGF2 affinities in the same range as IR-A and this is also reflected in the activation of the receptor. We did not observe any significant differences in either affinity or activation potential between IR-A and IR-B K718P or between IR-B and IR-A P718K. Hence by changing the one amino acid at position 718 the affinity is changed which was reflected in activation potential. This support the hypothesis that IR activation is mainly affinity dependent and no intrinsic differences in activation potential exist between IR-A and IR-B or between insulin, IGF1 and IGF2.

## Abbreviations

^125^I-Insulin: ^125^I-(Tyr31)-Insulin
ANOVA: one-way analysis of variance
BHK: baby hamster kidney
IGF: insulin-like growth factor
IGF1: insulin-like growth factor 1
IGF1R: human insulin-like growth factor 1 receptor
IGF2: insulin-like growth factor 2
IGFBPs: IGF binding proteins
IR: human insulin receptor
IR-A: human insulin receptor isoform A
IR-B: human insulin receptor isoform B
nM: nanomolar
PBS: phosphate buffered saline
pM: picomolar
SD: Standard deviation
SEM: Standard error of the mean
SPA: scintillation proximity assay
WGA: wheat germ agglutinin
WT: Wild type
